# The effectiveness of shared decision-making followed by positive reinforcement on physical disability in the long-term follow-up of patients with nonspecific low back pain in primary care: a clustered randomised controlled trial

**DOI:** 10.1186/s12875-018-0776-8

**Published:** 2018-06-28

**Authors:** Ariëtte R. J. Sanders, Jozien M. Bensing, Tessa Magnée, Peter Verhaak, Niek J. de Wit

**Affiliations:** 1 Julius Centre for Health Sciences and Primary Care, University Medical Centre Utrecht, PO Box 85500 3508, GA Utrecht, the Netherlands; 2 NIVEL (Netherlands Institute for Health Services Research), PO Box 1568 3500, BN Utrecht, the Netherlands; 3 Faculty of Social and Behavioural Science, Utrecht University, Utrecht, the Netherlands

**Keywords:** Low back pain, General practice, Patient-oriented outcome, Shared decision-making, Randomised controlled trial

## Abstract

**Background:**

Although the recovery of patients suffering from low back pain is highly context dependent, patient preferences about treatment options are seldom incorporated into the therapeutic plan. Shared decision-making (SDM) offers a tool to overcome this deficiency. The reinforcement by the general practitioner (GP) of a ‘shared’ chosen therapy might increase patients’ expectations of favourable outcomes and thus contribute to recovery.

**Methods:**

In the Netherlands, a clustered randomised controlled trial was performed to assess the effectiveness of shared decision-making followed by positive reinforcement of the chosen therapy (SDM&PR) on patient-related clinical outcomes. Overall, 68 GPs included 226 patients visiting their GP for a new episode of non-chronic low back pain. GPs in the intervention group were trained in implementing SDM&PR using a structured training programme with a focus on patient preferences in reaching treatment decisions. GPs in the control group provided care as usual. The primary outcome was the change in physical disability measured with the Roland-Morris disability questionnaire (RMD) during the six-month follow-up after the first consultation. Physical disability (RMD), pain, adequate relief, absenteeism and healthcare consumption at 2, 6, 12 and 26 weeks were secondary outcomes. A multivariate analysis with a mixed model was used to estimate the differences in outcomes.

**Results:**

Of the patients in the intervention and the control groups, 66 and 62%, respectively, completed the follow-up. Most patients (77%) recovered to no functional restrictions due to back pain within 26 weeks. No significant differences in the mean scores for any outcome were observed between intervention patients and controls during the follow-up, and in multivariate analysis, there was no significant difference in the main outcome during the six-month follow-up. Patients in the intervention group reported more involvement in decision-making.

**Conclusion:**

This study did not detect any improvement in clinical outcome or in health care consumption of patients with non-chronic low back pain after the training of GPs in SDM&PR. The implementation of SDM merely introduces task-oriented communication. The training of the GPs may have been more effective if it had focused more on patient-oriented communication techniques and on stressing the expectation of favourable outcomes.

**Trial registration:**

The Netherlands National Trial Register (NTR) number: NTR1960. The trial was registered in the NTR on August 20, 2009.

**Electronic supplementary material:**

The online version of this article (10.1186/s12875-018-0776-8) contains supplementary material, which is available to authorized users.

## Background

Nonspecific low back pain is defined as back pain localised below the costal margin and the inferior gluteal folds, with or without referred leg pain, and without a specific somatic origin [1, 2].

Low back pain can be divided into acute, with a duration of complaints < 6 weeks, subacute, with a duration of complaints between 6 and 12 weeks, and chronic, with complaints lasting longer than 3 months [1–3].

Low back pain has a lifetime prevalence of 60–85% [4]. Most episodes of low back pain resolve after two weeks, but the recurrence rate is high; three-quarters of patients have a second episode within one year [4]. Because of related health costs, absenteeism and disability, low back pain is a substantial economic burden to society [2, 5].

The therapeutic guidelines on low back pain focus on the continuation of physical activity, as the effectiveness of most therapeutic interventions does not exceed the placebo effect [1]. In addition, the guidelines recommend considering patient preferences in the choice of the therapeutic regimen because contextual factors determine the speed of recovery [1, 6]. Contextual factors include the patient, the physician, and their relationship [6].

The illness perceptions of a patient, such as avoidance beliefs and fear of the duration of the illness, predict the patient’s recovery and their return to work [7–9]. In medical decision-making, little attention is paid to the patient perspective, and even if considered, it is often misinterpreted [9–11]. However, the patient perspective is generally considered essential for medical decision-making, as stated in the Salzburger Statement on shared decision-making (SDM) [12].

SDM is defined as follows: A situation in which the professional and patient share their perspective and jointly decide on a treatment plan. SDM provides the possibility of incorporating patient preferences into clinical decision-making [13]. The philosophy of this concept is that patients will have more autonomy in decisions about their personal health if the doctor-patient relationship shifts from paternalistic to a more equal relationship [14]. Glyn Elwyn operationalised this concept into a three-talk model of shared decision-making. Team talk places an emphasis on the need to provide support to patients when they are made aware of choices, and Option talk refers to the task of comparing alternatives by using risk communication principles. Decision talk refers to the task of arriving at decisions that reflect the informed preferences of patients, guided by the experience and expertise of health professionals. In this broadly accepted model, patients are informed about the decision process and the pros and cons of treatment options [15].

Since the introduction of SDM in clinical care, research has focused on the process of SDM implementation and its effect on clinical outcomes [16]. At present, the findings related to clinical outcomes are scarce and unconvincing [16].

For patients with low back pain, SDM could improve the prognosis if patients were more adherent to treatment, as the expectation of a favourable outcome is incorporated into the treatment decisions [17].

It has been empirically proven that the positive outcome expectations of the patient benefit the health status of the patient, and the reinforcement of these treatment expectations could endorse these effects [18, 19].

Although widely advocated in guidelines, the effectiveness of SDM in the management of low back pain has not been evaluated in general practice [20].

Therefore, we conducted a large randomised controlled trial among primary care patients with non-chronic low back pain in the Netherlands and report the effectiveness of SDM followed by positive reinforcement of the therapeutic choice (SDM&PR) on recovery and healthcare consumption.

## Methods

### Aim

The aim is to assess the effectiveness of shared decision-making followed by a positive reinforcement of the chosen therapy (SDM&PR) on patient-related clinical outcomes in patients with non-chronic low back pain in general practice.

### Design and setting

A cluster-randomised controlled trial was performed in the practices of 68 general practitioners (GPs) in the academic primary care network around Utrecht in the Netherlands.

### Participants

GPs were recruited between August 2009 and May 2011. Each participating GP was requested to include ten patients with non-chronic nonspecific low back pain.

The inclusion criteria were as follows:

1. between 18 and 65 years of age, and
2. in consultation for a new episode of non-chronic nonspecific low back pain (as defined by the guidelines of the Dutch College of General Practitioners and the Cochrane Collaboration) [2, 21].

The exclusion criteria were as follows:

1. duration of low back pain longer than three months,
2. any previous episode of low back pain within the three months prior to the onset of the present episode,
3. pregnancy, and
4. insufficient mastery of the Dutch language.

Because the causes and pathophysiology of low back pain might be different in patients younger than 18 or older than 65 years, those who are pregnant or in those with a longer disease duration, we excluded these patients [1, 21].

### Randomisation, data collection and blinding

GPs were randomly assigned to the usual care (UC) group or the intervention (IV) group immediately after consenting to participate in the trial. Randomisation was done by research staff members who were not otherwise in the research project. Allocation was blinded using allocation cards in sealed envelopes in an initial block of 40 followed by blocks of ten envelopes. GPs in the control group were kept unaware of the communicative techniques that were trained. Auxiliary staff members recruited the patients. Patients and auxiliary practice staff members were not informed about the allocation of the GP or about the communicative techniques in the training programme. Auxiliary practice staff members collected questionnaires from the patients after inclusion. A follow-up questionnaire with a pre-paid envelope was given to each patient with instructions on when to complete it and send it to the research team. Patients were reminded to send the questionnaires two, six, twelve and twenty-six weeks after the consultation by email or phone just before the correct time and, if necessary, again two weeks later.

### Intervention

GPs in the intervention group were trained to perform SDM&PR during their consultations with the included patients. SDM followed the following process steps: inform the patient about therapeutic options, discuss the patient’s preferences, concerns and expectations, confirm the patient’s understanding, assess the patient’s preferred level of involvement in decision-making and finally make a joint decision about the optimal therapeutic regimen. GPs were trained to positively reinforce treatment outcomes after SDM.

### Training

GPs in the intervention group received two training sessions of two and a half hours. Training sessions were held in small groups of approximately three to five participants and were given by a peer GP with expertise in training SDM skills (AS).

The training was based on the learning principles of Kolb and the behavioural process elements of Elwyn [22]. To support SDM performance during consultations, the participating GPs received a desktop card summarising all consecutive process elements for SDM and a decision aid specifically developed for this trial according to the International Patient Decision Aids Standards (IPDAS)-guidelines (Additional file 1 Appendix 1) [23, 24]. Finally, they received individual feedback on their SDM performance based on observation by the trainer (AS) of videotapes of the consultation of each included patient. Details of the training are reported elsewhere [23]. The fidelity of the intervention was checked by measuring behavioural changes and consultation duration differences between the intervention and the control group using the OPTION instrument on videotaped consultations [23].

### Control group

In the control group, the GPs provided the usual standard of care. Although routine management was not predefined in the instructions for the study, GPs in the Netherlands are reported to follow the professional guidelines on low back pain in 70% of patients [25]. Discussion of the favourable prognosis of low back pain is part of the suggested management in the guideline, but SDM is not [2].

### Outcome

The primary outcome was the difference between the intervention and the control group in the course of functional disability during the six-month follow-up. Functional disability was measured daily during the first two weeks and at two, six, twelve and twenty-six weeks after the first consultation.

As secondary outcomes, we assessed the difference in functional disability at the time of each of the separate measurements (2, 6, 12 and 26 weeks), the difference in severity of back pain and the percentage of patients with adequate relief on separate measurement dates and at the end of the study.

As indicators of economic effect, we evaluated the differences in absenteeism and health care consumption between groups over the complete study period and on the separate measurement dates.

To be able to test for potential confounding, we measured illness perceptions at the baseline. To check the fidelity of the intervention, we questioned patients after the consultation about the level of involvement in decision-making.

### Measurements and instruments

Functional disability was assessed by the Dutch version of the Roland-Morris disability questionnaire (RMD). This validated questionnaire contains 24 closed questions about restrictions in daily activities during the previous day. The score is the total number of positive answers [26]. Pain severity was quantified by the validated continuous visual analogue scale (VAS), in which the patients indicate the level of pain during the past week on a continuous line that ranges from zero (no pain) to ten cm (the most terrible pain I can imagine), with outcomes measured in mm. [27]. Adequate relief (AR) of pain was assessed with one closed question referring to the recovery experienced since the previous questionnaire and was expressed as the percentage of patients with AR in each group.

Absenteeism was measured by the response to a question referring to the time before the baseline or since the previous questionnaire: ‘Because of my back pain, I refrained from work (absenteeism),’ yes/no/not applicable (expressed as a percentage of patients), followed by an inquiry about the number of days of sick leave.

Healthcare consumption data were derived from patient questionnaires by counting follow-up contacts, via telephone or at the practice, and expressed as the mean number of contacts per patient since the previous questionnaire.

Illness perceptions were assessed by the Dutch version of the abbreviated illness perception questionnaire (IPQ) [28]. This instrument measures eight separate dimensions of perceptions about low back pain using a scale rating from zero to ten. This instrument has been proven to be valid [29].

The actual level of shared decision-making, as experienced by the patient, was evaluated by their response to one simple question immediately after the consultation: ‘Were you involved in decision-making?’ Mean scores were calculated from a range of one to four points corresponding to the answers ‘no’, ‘mostly no’, mostly yes’ or ‘yes’.

The observed effects of the training were reported in a separate article on the evaluation of the training [23].

**Fig. 1:**
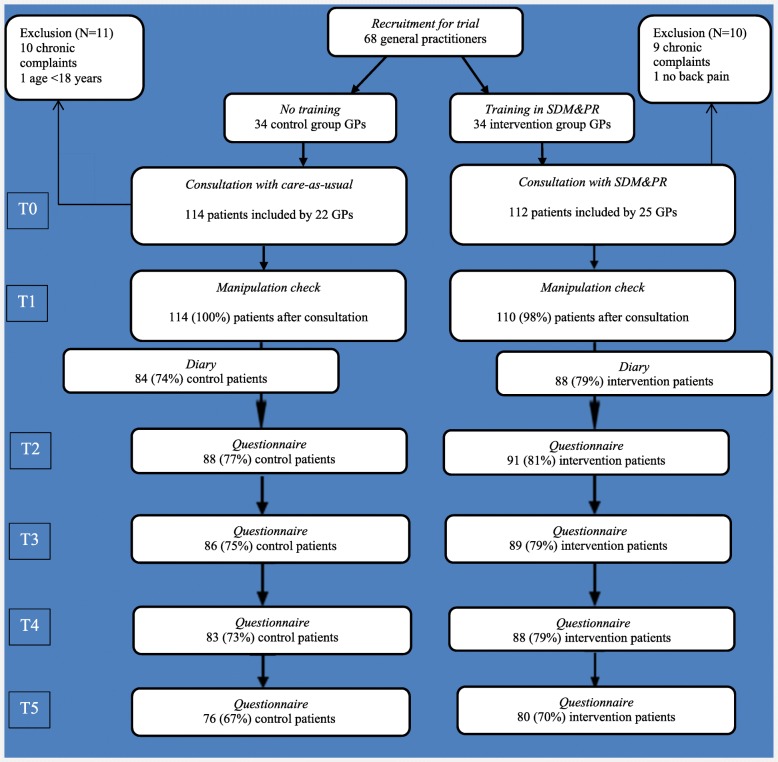
Flow chart of the participants in different phases through the trial. T0 = directly *before* the consultation; T1 = directly *after* the consultation; T2 = 2 weeks after consultation; T3 = 6 weeks after consultation; T4 = 12 weeks after consultation; T5 = 26 weeks after consultation

The primary outcome (RMD) and the VAS was assessed daily during the first 14 days by a diary and at two, six, twelve and twenty-six weeks after consultation by questionnaires. All other secondary outcomes were assessed at two, six, twelve and twenty-six weeks (Fig. 1). Baseline measurements, potential confounders and the manipulation check were assessed through questionnaires completed by all patients before and immediately after the consultation.

### Sample size

To reach a minimum standardised difference of 0.3 in the primary outcome between the intervention and control groups, which is more than 1 point on RMD scores with a standard deviation of 5, using a beta of 0.80 and an alpha of 0.05, 352 patients would be required [26]. As we randomised at the level of the GP but measured patient outcomes, we controlled for clustering effects. Based on clustering effects reported in earlier trials, we applied an intra-class correlation of 0.03 [30]. Presuming a 10% dropout rate, we calculated that 426 patients should be included by 60 GPs.

### Statistical analysis

Differences in baseline characteristics between dropouts and patients who completed the follow-up were tested for significance using a t-test for continuous variables and a X^2^-test for dichotomous and categorical variables.

The effect of the intervention on the separate measurement dates at two, six, twelve and twenty-six weeks was tested univariately. In multivariate analysis, differences in primary and secondary outcomes were estimated with a mixed model corrected for potential confounders: age, sex, educational level and the corresponding baseline value of the outcome. A random intercept was included for clustering at the level of the GP, and a random intercept and a random effect for time at the patient level were included to incorporate the effect over time. All analysis was performed on an intention-to-treat basis.

The potential confounding effect of each of the illness perception dimensions was assessed with mixed models, with restrictions at 12 weeks as the outcome variable and correction for all confounders.

Mixed models are robust for individual patients with missing follow-up measurements. In the analysis, we originally included the baseline measurement of the corresponding outcome as a covariate. Consequently, any measurement of any patient with a missing baseline measurement would be excluded from the analysis, thus reducing power and potentially introducing bias. We therefore decided to use multiple imputation to impute baseline variables and missing confounders measured at the baseline [31, 32].

Age, sex, educational level, absenteeism, all illness perceptions, the treatment allocation and all baseline measurements of primary and secondary outcomes were included in the multiple imputation. The numbers of imputed missing variables per baseline variable are described in Table 1. Five imputed datasets were created. Univariate and multivariate analyses were performed on each imputation; the results reported here were combined with Rubin’s rule [33].

**Table 1:** Baseline demographic and clinical characteristics of patients *in the complete* dataset. Continuous variable values are represented as means (standard deviation). Dichotomous variable values are represented as numbers (percentages)

	intervention group	control group
Patient characteristics	(*n* = 112)	(*n* = 114)
mean age (years)	45.4 (13.2)	44.3 (14.4)
male†	52 (47%)	55 (49%)
Dutch origin‡	97 (91%)	103 (93%)
educational level‡		
primary only	15 (14%)	19 (17%)
secondary	56 (52%)	53 (48%)
college, university	36 (34%)	39 (35%)
employed§	73 (70%)	71 (70%)
Baseline clinical characteristics		
functional disability score (RMD 0–24) (primary measure)¶	10.7 (5.0)	10.3 (5.2)
pain severity at baseline (VAS 0–100 mm)||	48.6 (16.0)	46.7 (16.7)
absenteeism (yes/no)||	39 (35%)	19 (20%)
illness perception dimensions (IPQ)(0–10)§		
consequences	6.3 (2.3)	6.1 (2.5)
timeline	4.2 (2.8)	3.5 (2.4)
personal control	5.0 (2.2)	5.4 (2.1)
treatment control	6.6 (1.9)	6.9 (1.9)
identity	6.9 (1.6)	7.2 (1.6)
concerns	4.5 (2.5)	4.8 (2.6)
illness comprehensibility	6.0 (2.3)	6.1 (2.3)
emotional response	5.0 (2.5)	5.2 (2.6)

RMD = Roland-Morris disability questionnaire (a higher score indicates a more favourable outcome). VAS = visual analogue scale combined score of low back pain, leg pain and both (a lower score indicates a more favourable outcome). IPQ = Illness Perception Questionnaire. ¶ *n* = 3 missing. || *n* = 34 missing. † *n* = 4 missing. ‡ *n* = 8 missing. § *n* = 19 missing

## Results

### Participants

Sixty-eight GPs agreed to participate and were randomised to the intervention (*n* = 34) or the control group (*n* = 34). GPs in the intervention group did not differ from control GPs with regard to sex, age, professional age, number of included patients or percentage of GP trainers per group.

Between January 2010 and January 2012, forty-seven GPs included 247 patients (range 0–10). Twenty-one of these patients did not meet the inclusion criteria because they did not align with the definition of non-chronic low back pain (*n* = 19), did not have back complaints (*n* = 1) or were younger than 18 years old (*n* = 1). Ultimately, 114 patients in the control group and 112 in the intervention group were included in the analysis (Fig. 1).

Patients in the intervention and control groups were comparable across most baseline measurements (Table 1 and Additional file 2 Appendix 2 for the imputed dataset). Patients in the intervention group reported more absenteeism from work due to their low back pain, and they more frequently expected their pain to last longer than did the controls.

During the follow-up, 76 (67%) of the control patients and 80 (70%) of the intervention patients completed all questionnaires. Overall, 71 (62%) patients in the control group and 75 (66%) patients in the intervention group completed the diary and all questionnaires (Fig. 1). Patients who did not complete the follow-up were more frequently of non-Dutch origin (15% non-Dutch natives in dropouts versus 5% non-Dutch natives in the analysed group; *p* = 0.017) and were younger (a mean age of 39.2 years for dropouts versus 47.4 years for the analysed group; *p* = 0.000). They did not differ significantly in other baseline measurements.

### Intervention effect

**Fig. 2:**
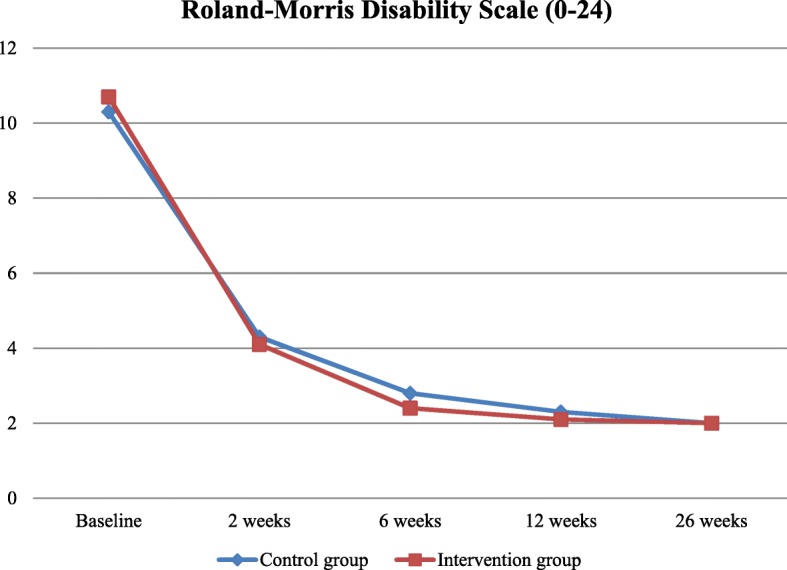
Patients experiencing disabilities after 2, 6, 12 and 26 weeks. Observed mean scores in experienced disabilities (measured by RMD scale) per group without correction for clustering between baseline and 26 weeks in time. At baseline, 226 patients, at 2 weeks, 179 patients, at 6 weeks, 175 patients, at 12 weeks, 171 patients and at 26 weeks, 156 patients. Minimal clinically important change = 3.5 [34]

The mean disability score among the patients in the intervention and the control groups declined to 4.1 (SDM&PR group) and 4.3 (control group) after 2 weeks (difference 0.2; *p*-value 0.789), 2.1 (SDM&PR) and 2.3 (controls) after 12 weeks (difference 0.2; *p*-value 0.720) and 2.0 for both groups after 26 weeks (difference 0.0; p-value 0.949) (Table 2 and Fig. 2).

**Table 2:** Univariate mean score per group in primary and secondary outcomes in the imputed dataset without correction for clustering

	MEAN SCORE AT 2 WEEKS			Mean score at 12 weeks			mean score at 26 weeks		
	IV	CO	*p*-value	IV	CO	*p*-value	IV	CO	*p*-value
*Clinical parameters*									
Disability (RMD) (0–24)	4.1 (5.3)	4.3 (4.8)	0.789	2.1 (4.0)	2.3 (3.7)	0.720	2.0 (3.7)	2.0 (3.6)	0.949
Pain (VAS) (0–100 mm)	18.9 (21.7)	20.3 (20.9)	0.675	14.2 (22.6)	12.4 (17.5)	0.577	13.6 (17.3)	16.3 (21.2)	0.385
Adequate relief (yes/no)†	70 (81%)	62 (81%)	0.888	45 (69%)	38 (62%)	0.416	35 (66%)	32 (64%)	0.830
*Societal impact*									
Absenteeism (days)	1.47 (3.35)	2.05 (4.03)	0.359	0.93 (5.65)	0.800 (3.63)	0.888			*
Absenteeism (yes/no)‡	18 (28%)	20 (27%)	0.924	3 (5%)	4 (8%)	0.552	7 (11%)	6 (14%)	0.650
*healthcare consumption*									
Telephone consultations (per patient)	0.35 (0.71)	0.29 (0.60)	0.556	1.18 (0.50)	1.18 (0.51)	0.672			*
Practice consultations (per patient)	0.21 (0.51)	0.11 (0.39)	0.134	1.12 (0.43)	1.15 (0.52)	0.965	1.11 (0.45)	1.10 (0.38)	0.914

RMD = Roland-Morris disability questionnaire (a higher score indicates a more favourable outcome). VAS = visual analogue scale (a lower score indicates a more favourable outcome). Mean score of low back pain, leg pain and both. IPQ = illness perception questionnaire. CO = control group. IV = intervention group. *Cannot be computed because the group is zero

The mean pain score in the two groups was 18.9 (SDM&PR) and 20.3 (controls) after 2 weeks (difference 1.4; *p*-value 0.675), 14.2 (SDM&PR) and 12.4 (controls) after 12 weeks (difference 1.8; p-value 0.577) and 13.6 (SDM&PR) and 16.3 (controls) after 26 weeks (difference 2.7; p-value 0.385). The percentage of patients with adequate relief was 70% (SDM&PR) and 62% (controls) after 2 weeks (p-value 0.888), 49% in both groups after 6 weeks, 69% (SDM&PR) and 62% (controls) after 12 weeks, and 66% (SDM&PR) and 64% (controls) after 26 weeks. The mean number of days of absenteeism and mean health care consumption at 2, 6, 12 and 26 weeks did not differ between the two groups (Table 2).

In the multilevel, multivariate analysis, correcting for baseline differences, patient characteristics and the clustering effect, the mean difference in disability scores between the intervention and control groups during the six-month follow-up was − 0.259 (*p*-value 0.582) (Table 3). The mean difference in pain score between the two groups in the six-month follow-up was − 2.269 (p-value 0.306). During the follow-up, the two groups did not differ in the percentage of patients with adequate relief, the number of days of absenteeism or in healthcare consumption (Table 3).

**Table 3:** Difference in mean scores between the control and intervention groups *in the imputed dataset* during the six-month follow-up

	UNIVARIATE ANALYSIS*			multivariate analysis	
	mean difference/rate ratio	CI	*p*-value	mean difference/ rate ratio	*p*-value
ENDPOINT					
Disability (RMD) (scale 0–24) ¶	−0.233	−1.258 to 0.791	0.655	−0.259	0.582
*Secondary outcomes*					
Pain (VAS) (scale 0–100 mm) ¶	−1.120	−6.133 to 3.893	0.662	−2.269	0.306
Adequate relief (yes/no) †	1.118	0.510–1.567	0.696	1.119	0.730
Absenteeism (in days) ‡	1.032	0.927–1.338	0.249	0.332	0.506
*Healthcare consumption*					
Telephone consultations (number per patient) §	1.0142	1.001–1.018	0.845	1.020	0.789
Practice consultations (number per patient) §	1.0143	0.880–1.169	0.845	1.067	0.245

RMD = Roland-Morris disability questionnaire (a higher score indicates a more favourable outcome). VAS = visual analogue scale (a lower score indicates a more favourable outcome). Mean score of low back pain, leg pain and both. *Corrected for clustering effect. ¶ Mean difference between control and intervention groups over 26 weeks. † Odds ratio without baseline correction. ‡ Rate ratio in the multilevel model corrected for dichotomous baseline value. § Rate ratio without baseline correction

Of the 8 dimensions of illness perception, only consequences (β = 1.24 confidence interval (CI) 0.14–2.35), timeline (β = 1.38 CI 0.27–2.48) and concern (β = 1.45 CI 0.34–2.56) were significantly associated with disability at 12 weeks. However, when the interaction term of each of these three items with the intervention was added to the multivariate model, no significant effect of illness perception on disability at 12 weeks was found.

In both groups, patients reported a substantial degree of involvement in decision-making. However, the patients in the intervention group reported a significantly higher level of patient involvement (2.92 standard deviation (SD) 1.21; range 1–4) than the controls (2.44 SD 1.23) (difference 0.48; *p*-value 0.005).

When studying the fidelity of the intervention, we measured significant differences in the SDM behaviour in favour of the intervention group and a mean duration of the consultation of 16 min for the intervention group versus 13 min for the control group [23].

## Discussion

This training of general practitioners in SDM&PR did not improve the symptom recovery of patients with non-chronic low back pain in primary care, even though the GPs effectively involved patients in the choice of treatment after the training. At no point in the follow-up did the mean disability or pain score of the patients whose GP was trained in SDM&PR differ from those patients whose GP provided the usual standard of care (Fig. 2). Patients in both groups reported that pain and physical limitations gradually declined and returned to a normal population level at 26 weeks [34].

The comparable clinical discourse in the two groups was also reflected in pain-related absenteeism from work and in health care consumption during the follow-up.

Patients who attributed much importance to the consequences of their back pain, those who expected the pain to last long and those who had many concerns about the pain had a poorer prognosis for symptom recovery. However, the prognosis was independent of the performance of SDM&PR by the GPs.

### Strengths of the study

Most research on SDM thus far has focused on process outcomes and not on patient-related clinical outcomes [20].

We performed a randomised controlled trial among patients with low back pain recruited in daily primary care and evaluated the effectiveness of SDM on relevant clinical outcomes. Based on current knowledge, we constructed a multifaceted intervention and training programme that was grounded in a theoretical concept of SDM, involving both participants in the decision process [20].

Participating GPs were well trained, and the positive SDM performance during consultations after the training was acknowledged by the patients [23]. We used a mixed model analysis because these are robust for individual patients with missing follow-up measurements under the assumption of missing values completely at random or missing values at random dependent on another variable included in the mixed model. As in most studies, the correctness of this assumption of ‘random missing’ cannot be proven in our study. In our view, however, it is very unlikely that a treatment effect was not observed due to the multiple imputation procedure because the complete case analyses confirmed the lack of a treatment effect (Additional file 3 Appendix 3).

### Limitations of the study

The patient recruitment met only half (53%) of the pre-set sample size. Participating GPs experienced problems that hampered recruitment, possibly due to unforeseen changes in the healthcare system, such as the introduction of direct access physiotherapy. However, the fact that the results of the patients in the intervention and control groups did not differ in any of the outcome measures at any moment in time demonstrates in our view the consistency of the results, and even if differences were demonstrated at the pre-set sample size, these differences would have been small and of questionable clinical relevance.

Dropout rates of GPs and patients are similar in the intervention and control groups and are in line with other studies on patients suffering from nonspecific low back pain in primary care in the Netherlands [35]. We cannot think of any reason why the intervention should have influenced the dropout rate of patients, but we estimate that dropout is not related to the intervention but is rather related to the complex disease course of nonspecific low back pain and the mismatch between patient expectations and the professional’s management.

Although we have observed significant differences in the perception of SDM between the experimental groups, we believe that the difference should be evaluated in the context of treatment fidelity. In a recent publication evaluating the intervention from an observational perspective, we found significant differences in the use of physical examination and in the consultation duration between groups [23]. Moreover, we question whether the patient perspective was sufficiently considered to incorporate the patient’s positive expectations into the actual decision despite significant differences in the SDM behaviour of GPs between the groups.

Unfortunately, as with many other studies on the effects of SDM on health outcomes, the effects are too small to allow conclusions about the impact of the separate communicative techniques, SDM and positive reinforcement of treatment expectations. Theoretically, we expected a positive interaction between the two techniques. The consistent pattern of very small potential positive treatment effects of the combined intervention above the usual standard of care could be explained by a stronger positive effect of one technique counteracted by the effect of the other (Fig. 2). For instance, in a study by a physiotherapist on the effects of SDM on the prognosis of low back pain, even negative expectations of patients were suggested to be responsible for poorer health outcomes after SDM than the usual standard of care [36]. Conversely, in a study evaluating the placebo effect on chronic low back pain, positive treatment expectations and a supportive environment were considered responsible for short-term relief from complaints [16, 23].

Studies on the effects of learned communicative strategies frequently face problems with blinding. We downsized the risk of non-blinding by sorting patients per GP, by recruiting patients via auxiliary staff members unaware of the allocation, and by not providing details of the trained communicative strategy to control GPs, auxiliary staff members and patients.

We did not perform a full health economic assessment but restricted the economic impact analysis to measuring the absenteeism of workers. However, in a detailed cost-effectiveness analysis, the reported 20% difference in the duration of the consultation time should be considered [23].

### Possible explanations

As in other studies on interventions for non-chronic low back pain, we did not find substantial or significant effects [37].

This finding could be attributed to different factors. Such as an excessively diverse study population in the duration of the complaints or in patient characteristics [36–38].

Although we think that the risk of contamination was limited, GPs in the control group may have incorporated SDM in their consultations as well. This might be reflected in the fact that the mean score for the question of whether patients felt involved in the decision-making was between ‘mostly yes’ and ‘mostly no’. The difference in the results of the intervention group, where the mean score was ‘mostly yes’, was significant but limited. However, the observational study demonstrates low levels of SDM in both groups despite the significant effect of training in SDM behaviour [11, 23, 39, 40].

Because most patients quickly recover from their back pain, the intervention simply might not have had sufficient discriminative content above the spontaneous course. In their review of psychosocial interventions for non-chronic low back pain in primary care, Ramond et al. [38] advise the integration of several psychosocial factors with multicomponent interventions to overcome this problem.

Contextual factors play an important role in the symptom perception, prognosis and recovery of low back pain [1, 4]. We identified three subgroups of patients with a poorer prognosis for symptom recovery in the analysis of the effect of each of the illness perception dimensions on the restrictions at twelve weeks. Patients with more negative illness perceptions or with a longer duration of complaints before they contact their GP might be better helped by more positive treatment expectations, but unfortunately, our dataset did not allow subgroup analysis on the effect of the intervention for particular patient characteristics [38]. Differences between the contextual factors of patients in the intervention and control groups may have influenced the results. Although the patients were not randomised, we have no indication that the recruitment to the intervention and control groups resulted in selection bias [18, 28, 41, 42].

Although the GPs were extensively trained before participating in the intervention group and the patients recognised SDM during the actual consultations, we question, based on the results of the evaluation of the training, whether the training did result in adequate SDM performance [40, 43].

Trained GPs became more aware of the need to better inform patients about treatment options and to incorporate patients’ expectations during the intake phase of the consultation, but they persisted in providing a paternalistic, guideline-oriented choice.

In a review on the effects of the implementation of SDM in clinical encounters measured by an external observer, Couët et al. [40] reported similar training effects and noticed that only incidentally is clinical management adjusted to patient preferences. In the evaluation of the training, we confirm this observation and conclude that patient preferences were insufficiently considered in the actual decision-making to incorporate the patient’s positive treatment expectations into the treatment choice [23]. When patient preferences are not reflected in treatment choices, the impact of positive reinforcement of the therapeutic plan on patient recovery will diminish.

So far, task-oriented issues, such as performing process steps and information exchange, are emphasised in the implementation of SDM [20]. However, the effects of knowledge transfer on proportional understanding are questionable, and the effects on recovery are unclear [20, 43].

Future research on the involvement of patients in treatment decisions should therefore focus more on professional attitude and equality in the patient professional relationship as a condition for successful SDM.

## Conclusion

Training of GPs in the application of SDM&PR during consultations with patients with non-chronic low back pain did not significantly improve clinical recovery. Although it may have improved the ‘knowledge and rationalise expectations’ of the patients, this did not lead to less functional impairment, shorter pain duration or less absenteeism from work than routine practice. Most patients recovered from their low back-pain within 12 weeks, and this positive effect was persistent at the 26-week follow-up, which confirms the benign natural course of low back-pain as reported in the literature. A potential small positive effect of either SDM or positive reinforcement of treatment expectations cannot be excluded. As the prognosis of low back pain is predominantly determined by psychosocial factors, we suggest that further research on the positive health effects of communicative techniques should focus on a more patient-oriented approach, combined with the reinforcement of positive recovery expectations, than on task-oriented techniques such as SDM.

## Additional files

Additional file 1: **Appendix 1.** Desktop tool translated Original Dutch version and translation of the desktop tool (DOCX 17 kb)

Additional file 2: **Appendix 2.** BT imputed Baseline Table of imputed dataset Baseline demographic and clinical characteristics of patients in imputed dataset. (DOCX 16 kb)

Additional file 3: **Appendix 3.** Complete case analysis. Difference in mean scores between the control and intervention group during the six-month follow-up in complete cases. (DOCX 18 kb)

## Abbreviations

AR: Adequate relief
AS: Ariëtte Sanders
CI: Confidence interval
GP: General practitioner
IPDAS: International Patient Decision Aids Standards
IPQ: Illness perception questionnaire
IV: Intervention
NTR: The Netherlands National Trial Register
RMD: Roland-Morris disability questionnaire
SD: Standard deviation
SDM: Shared decision-making
SDM&PR: Shared decision-making followed by a positive reinforcement of the chosen therapy
UC: Usual care
VAS: Visual analogue scale

## References

[1] van Tulder M, Becker A, Bekkering T, Breen A, del Real MT, Hutchinson A (2006). Chapter 3. European guidelines for the management of acute nonspecific low back pain in primary care. Eur Spine J.

[2] Bons SCS, Borg MAJP, Van den Donk M, et al. NHG-Standaard Aspecifieke lagerugpijn (Tweede herziening). In: Richtlijnen en praktijk. Het Nederlands Huisartsen Genootschap. 2017. https://www.nhg.org/standaarden/volledig/nhg-standaard-aspecifieke-lagerugpijn. Accessed 20 June 2018.

[3] Dionne CE, Dunn KM, Croft PR, Nachemson AL, Buchbinder R, Walker BF (2008). A consensus approach toward the standardization of back pain definitions for use in prevalence studies. Spine.

[4] Krismer M, van Tulder M (2007). Strategies for prevention and management of musculoskeletal conditions. Low back pain (non-specific). Best Pract Res Clin Rheumatol.

[5] Dagenais S, Caro J, Haldeman S (2008). A systematic review of low back pain cost of illness studies in the United States and internationally. Spine J.

[6] Di Blasi Z, Harkness E, Ernst E, Georgiou A, Kleijnen J (2001). Influence of context effects on health outcomes: a systematic review. Lancet.

[7] Iles RA, Davidson M, Taylor NF (2008). Psychosocial predictors of failure to return to work in non-chronic non-specific low back pain: a systematic review. Occup Environ Med.

[8] Iles RA, Davidson M, Taylor NF, O'Halloran P (2009). Systematic review of the ability of recovery expectations to predict outcomes in non-chronic non-specific low back pain. J Occup Rehabil.

[9] van Tol-Geerdink JJ, Stalmeier PF, van Lin EN, Schimmel EC, Huizenga H, van Daal WA (2006). Do patients with localized prostate cancer treatment really want more aggressive treatment?. J Clin Oncol.

[10] Bensing J (2000). Bridging the gap. The separate worlds of evidence-based medicine and patient-centered medicine Patient Educ Couns.

[11] Stalmeier PFM, van Tol-Geerdink JJ, van Lin ENJT, Schimmel E (2009). Huizenga H, van Daal WA, et al. [the patient chooses for feasibility and effectiveness]. NedTijdschrGeneeskd.

[12] Salzburg Global Seminar (2011). Salzburg statement on shared decision making. BMJ.

[13] Edwards A, Elwyn G (2006). Inside the black box of shared decision making: distinguishing between the process of involvement and who makes the decision. Health Expect.

[14] Stiggelbout AM, Van der Weijden T, De Wit MP (2012). Shared decision making: really putting patients at the Centre of healthcare. BMJ.

[15] Elwyn G, Durand MA, Song J (2017). A three-talk model for shared decision making: multistage consultation process. BMJ.

[16] Légaré F, Stacey D, Turcotte S, Cossi MJ, Kryworuchko J, Graham ID (2014). Interventions for improving the adoption of shared decision making by healthcare professionals. Cochrane Database Syst Rev.

[17] Stacey D, Légaré F, Lewis K, Barry MJ, Bennett CL, Eden KB (2011). Decision aids for people facing health treatment or screening decisions. Cochrane Database Syst Rev.

[18] Benedetti F, Amanzio M (2013). Mechanisms of the placebo response. Pulm Pharmacol Ther.

[19] Thomas KB (1987). General practice consultations: is there any point in being positive?. Br Med J (Clin Res Ed).

[20] Légaré F, Ratté S, Stacey D, Kryworuchko J, Gravel K, Graham ID (2010). Interventions for improving the adoption of shared decision making by healthcare professionals. Cochrane Database Syst Rev.

[21] Saragiotto BT, Machado GC, Ferreira ML, Pinheiro MB, Abdel Shaheed C, Maher CG (2016). Paracetamol for low back pain. Cochrane Database Syst Rev.

[22] Elwyn G, Hutchings H, Edwards A, Rapport F, Wensing M, Cheung WY, Grol R (2005). The OPTION scale: measuring the extent that clinicians involve patients in decision-making tasks. Health Expect.

[23] Sanders AR, Bensing JM, Essed MA, Magnée T, de Wit NJ, Verhaak PF (2017). Does training general practitioners result in more shared decision making during consultations?. Patient Educ Couns.

[24] Elwyn G, O'Connor AM, Bennett C, Newcombe RG, Politi M, Durand MA (2009). Assessing the quality of decision support technologies using the international patient decision aid standards instrument (IPDASi). PLoS One.

[25] Schers H, Wensing M, Huijsmans Z, van Tulder M, Grol R (2001). Implementation barriers for general practice guidelines on low back pain a qualitative study. Spine (Phila Pa 1976).

[26] Brouwer S, Kuijer W, Dijkstra PU, Göeken LN, Groothoff JW, Geertzen JH (2004). Reliability and stability of the Roland Morris disability questionnaire: intra class correlation and limits of agreement. Disabil Rehabil.

[27] Ostelo RW, de Vet HC (2005). Clinically important outcomes in low back pain. Best Pract Res Clin Rheumatol.

[28] Foster NE, Bishop A, Thomas E, Main C, Horne R, Weinman J (2008). Illness perceptions of low back pain patients in primary care: what are they, do they change and are they associated with outcome?. Pain.

[29] Broadbent E, Petrie KJ, Main J, Weinman J (2006). The brief illness perception questionnaire. J Psychosom Res.

[30] Jellema P, van der Windt DA, van der Horst HE, Blankenstein AH, Bouter LM, Stalman WA (2005). Why is a treatment aimed at psychosocial factors not effective in patients with (sub)acute low back pain?. Pain.

[31] Donders AR, van der Heijden GJ, Stijnen T, Moons KG (2006). A gentle introduction to imputation of missing values. J Clin Epidemiol.

[32] Sterne JA, White IR, Carlin JB, Spratt M, Royston P, Kenward MG (2009). Multiple imputation for missing data in epidemiological and clinical research: potential and pitfalls. BMJ.

[33] Rubin LH, Witkiewitz K, Andre JS, Reilly S (2007). Methods for handling missing data in the behavioral neurosciences: don't throw the baby rat out with the bath water. J Undergrad Neurosci Educ.

[34] Kovacs FM, Abraira V, Royuela A, Corcoll J, Alegre L, Cano A (2007). Minimal clinically important change for pain intensity and disability in patients with nonspecific low back pain. Spine (Phila Pa 1976).

[35] Jellema P, van der Windt DA, van der Horst HE, Twisk JW, Stalman WA, Bouter LM (2005). Should treatment of (sub)acute low back pain be aimed at psychosocial prognostic factors? Cluster randomised clinical trial in general practice. BMJ.

[36] Gysels M, Richardson A, Higginson IJ (2004). Communication training for health professionals who care for patients with cancer: a systematic review of effectiveness. Support Care Cancer.

[37] Pengel HM, Maher CG, Refshauge KM (2002). Systematic review of conservative interventions for subacute low back pain. Clin Rehabil.

[38] Ramond-Roquin A, Bouton C, Gobin-Tempereau AS, Airagnes G, Richard I, Roquelaure Y (2014). Interventions focusing on psychosocial risk factors for patients with non-chronic low back pain in primary care--a systematic review. Fam Pract.

[39] Schers H, Braspenning J, Drijver R, Wensing M, Grol R (2000). Low back pain in general practice: reported management and reasons for not adhering to the guidelines in the Netherlands. Br J Gen Pract.

[40] Couët N, Desroches S, Robitaille H, Vaillancourt H, Leblanc A, Turcotte S (2015). Assessments of the extent to which health-care providers involve patients in decision making: a systematic review of studies using the OPTION instrument. Health Expect.

[41] Benedetti F, Lanotte M, Lopiano L, Colloca L (2007). When words are painful: unraveling the mechanisms of the nocebo effect. Neuroscience.

[42] Patel S, Ngunjiri A, Hee SW, Yang Y, Brown S, Friede T (2014). Primum non nocere: shared informed decision making in low back pain--a pilot cluster randomised trial. BMC Musculoskelet Disord.

[43] Braddock CH (2010). The emerging importance and relevance of shared decision making to clinical practice. Med Decis Mak.

