# Supplementary material for: The effectiveness of shared decision-making followed by positive reinforcement on physical disability in the long-term follow-up of patients with nonspecific low back pain in primary care: a clustered randomised controlled trial
Source: BMC Fam Pract. 2018 Jun 28;19:102. doi: 10.1186/s12875-018-0776-8 (PMC6022513; doi:10.1186/s12875-018-0776-8)
Supplement: Supplementary file 1 — Appendix 1. Desktop tool translated Original Dutch version and translation of the desktop tool (DOCX 17 kb) [file 12875_2018_776_MOESM1_ESM.docx]

**Prompt sheet**

1. Voorkeuren van de patiënt voor de therapie gevraagd?
   1. *Wat zou u van mij willen?*
   2. *Wat had u er zelf van tevoren over bedacht?*
   3. *Wat denkt u zelf?*
   4. *Heeft u zelf een idee over de beste aanpak?*

Were the patients asked about their therapy preferences?

1. What do you expect of me?
2. What are your expectations of the therapy?
3. What are your own thoughts on the therapy?
4. Do you have ideas regarding the most suitable approach?
5. Verwachtingen van patiënt over therapie bekend
6. *Welke aanpak kent u?*
7. *Wat is uw ervaring/verwachting daarover?*
8. *Waar hoopt u op?*

Are the patients’ expectations about the therapy known?

1. What approaches are you aware of?
2. What are your experiences with those approaches?
3. What are your hopes for this approach?
4. Zorgen van patiënt over probleem uitgevraagd
5. *Wat dacht u toen u hier naartoe kwam?*
6. *Waar ligt uw zorg?*
7. *Bent u ergens bang voor?*

Were the patients questioned about their worries regarding their medical problems?

1. What were your thoughts on your way here?
2. What are your main concerns?
3. Are you afraid of anything specific regarding the therapy?
4. Keuzehulp uitgelegd en consequenties duidelijk aan patiënt
5. *Kunt u zo uw keuze maken*
6. *Helpt dit u bij het maken van een keuze?*

Were the available options and resulting consequences explained clearly to the patients?

1. Are you able to make your choice with the given information?
2. Does this explanation help you make a decision?
3. Ruimte gegeven aan patiënt om vragen te stellen
4. *Heeft u hier nog vragen over?*

Have the patients been given an opportunity to ask questions?

1. Do you have any questions?
2. Voorkeur rol patiënt m.b.t. maken van keuze helder
3. *Zou u zelf een keuze willen maken?*
4. *Of wilt u dat ik u daarbij help?*

Are the patients’ decision-making preferences clear?

1. Would you like to make the decision on your own?
2. Would you like me to help you make a decision?
3. Besluitvorming in gezamenlijk overleg
4. *Wat zou uw keuze zijn?*
5. *Wat zou u beslissen?*
6. *Waaraan geeft u de voorkeur?*

Shared decision-making

1. What is your choice?
2. What have you decided?
3. Which option do you prefer?
4. Verwachtingen van patiënt positief bekrachtigd
5. *Naar verwachting zal de pijn op korte termijn overgaan. Als u daarbij goed blijft bewegen zal dat zeker helpen.*
6. *U zou graag ……… willen, dat is een keus die bij u past. Dat zal u zeker helpen bij het herstel.*

Are the patients’ expectations positively reinforced?

1. My expectation is that the pain will go away quickly. By moving regularly, you can facilitate recovery.
2. You would like to ……., which is a suitable option for you. That will definitely help your recovery.
