# Supplementary material for: The effectiveness of shared decision-making followed by positive reinforcement on physical disability in the long-term follow-up of patients with nonspecific low back pain in primary care: a clustered randomised controlled trial
Source: BMC Fam Pract. 2018 Jun 28;19:102. doi: 10.1186/s12875-018-0776-8 (PMC6022513; doi:10.1186/s12875-018-0776-8)
Supplement: Supplementary file 2 — Appendix 2. BT imputed Baseline Table of imputed dataset Baseline demographic and clinical characteristics of patients in imputed dataset. (DOCX 16 kb) [file 12875_2018_776_MOESM2_ESM.docx]

Appendix 2

Table 1a Baseline demographic and clinical characteristics of patients *in imputed* dataset. Continuous variable values are represented as means (standard deviation). Dichotomous variable values are represented as numbers (percentages).

|  | intervention group | control group |
| --- | --- | --- |
| ***Patient characteristics*** | (n=112) | (n=114) |
| mean age (years) | 45.4 (13.2) | 44.3 (14.4) |
| male | 53 (47%) | 56 (49%) |
| Dutch origin | 102 (91%) | 106 (93%) |
| educational level |  |  |
| primary only | 15 (13%) | 19 (17%) |
| secondary | 60 (54%) | 53 (47%) |
| college, university | 37 (33%) | 42 (37%) |
| employed | 79 (71%) | 81 (71%) |
| ***Baseline clinical characteristics*** |  |  |
| functional disability score  (RMD 0-24) (primary measure) | 10.7 (5.0) | 10.3 (5.1) |
| pain severity at baseline  (VAS 0-100 mm) | 48.9 (15.4) | 46.4 (15.8) |
| absenteeism (yes/no) | 53 (47%) | 36 (32%) |
| illness perception dimensions  (IPQ) (0-10) |  |  |
| consequences | 6.2 (2.3) | 6.0 (2.5) |
| timeline | 4.2 (2.7) | 3.5 (2.4) |
| personal control | 5.0 (2.2) | 5.5 (2.0) |
| treatment control | 6.6 (1.9) | 6.9 (1.8) |
| identity | 6.9 (1.6) | 7.2 (1.5) |
| concerns | 4.5 (2.5) | 4.8 (2.6) |
| illness comprehensibility | 5.9 (2.3) | 6.2 (2.2) |
| emotional response | 5.0 (2.5) | 5.2 (2.6) |

RMD=Roland-Morris Disability questionnaire (a higher score indicates a more favourable outcome). VAS=visual analogue scale combined score of low back pain. leg pain and both (a lower score indicates a more favourable outcome). IPQ=Illness Perception Questionnaire.
