# Supplementary material for: The effectiveness of shared decision-making followed by positive reinforcement on physical disability in the long-term follow-up of patients with nonspecific low back pain in primary care: a clustered randomised controlled trial
Source: BMC Fam Pract. 2018 Jun 28;19:102. doi: 10.1186/s12875-018-0776-8 (PMC6022513; doi:10.1186/s12875-018-0776-8)
Supplement: Supplementary file 3 — Appendix 3. Complete case analysis. Difference in mean scores between the control and intervention group during the six-month follow-up in complete cases. (DOCX 18 kb) [file 12875_2018_776_MOESM3_ESM.docx]

Appendix 3 Please note the univariate analyses are not influenced by imputation as only baseline variables were imputed in the univariate analysis.

Table 3a Difference in mean scores between control and the intervention group during the six-month follow-up *on complete cases*.

|  | univariate analysis* |  |  | multivariate analysis |  |
| --- | --- | --- | --- | --- | --- |
|  | mean difference/rate ratio | CI | p-value | mean difference/rate ratio | p-value |
| ENDPOINT |  |  |  |  |  |
| Disability score  (RMD) (scale 0-24) (primary measure) ¶ | -0.233 | -1.258 to 0.791 | 0.655 | -0.495 | 0.280 |
|  |  |  |  |  |  |
| *Secondary outcomes* |  |  |  |  |  |
| Pain (VAS) (scale 0-100 mm) ¶ | -1.120 | -6.133 to 3.893 | 0.662 | -2.297 | 0.312 |
| Adequate relief (yes/no) † | 1.118 | 0.510-1.567 | 0.696 | 1.053 | 0.829 |
| Absenteeism (in days) ‡ | 1.032 | 0.927-1.338 | 0.249 | 0.889 | 0.769 |
| *Healthcare consumption* |  |  |  |  |  |
| Telephone consultations (number per patient) § | 1.0142 | 1.001-1.018 | 0.845 | 1.020 | 0.780 |
| Practice consultations (number per patient) § | 1.0143 | 0.880-1.169 | 0.845 | 0.999 | 0.989 |

RMD=Roland-Morris disability questionnaire (a higher score indicates a more favourable outcome). VAS=visual analogue scale (a lower score indicates a more favourable outcome). Mean score of low back pain, leg pain and both. * Corrected for clustering effect. ¶ Mean difference between control and intervention group over 26 weeks. † Odds ratio without baseline correction. ‡ Rate ratio in the multilevel model corrected for dichotomous baseline value. § Rate ratio without baseline correction. Multilevel: corrected for gender. age. educational level and clustering on GP level.
